# Visceral Obesity and Its Shared Role in Cancer and Cardiovascular Disease: A Scoping Review of the Pathophysiology and Pharmacological Treatments

**DOI:** 10.3390/ijms21239042

**Published:** 2020-11-27

**Authors:** Erika Aparecida Silveira, Golnaz Vaseghi, Annelisa Silva de Carvalho Santos, Nathalie Kliemann, Farzad Masoudkabir, Matias Noll, Noushin Mohammadifard, Nizal Sarrafzadegan, Cesar de Oliveira

**Affiliations:** 1 Department of Epidemiology & Public Health, Institute of Epidemiology & Health Care, University College London, London WC1E 6BT, UK; c.oliveira@ucl.ac.uk; 2 Postgraduate Program in Health Sciences, Faculty of Medicine, Federal University of Goiás, Goiânia 74690-900, Goiás, Brazil; annelisa.nut@gmail.com (A.S.d.C.S.); matias.noll@ifgoiano.edu.br (M.N.); 3 Applied Physiology Research Center, Cardiovascular Research Institute, Isfahan University of Medical Sciences, Isfahan 8158388994, Iran; golnazvaseghi@yahoo.com; 4 United Faculty of Campinas, Goiânia 74525-020, Goiás, Brazil; 5 Nutritional Epidemiology Group, Nutrition and Metabolism Section, International Agency for Research on Cancer, World Health Organization, 69372 Lyon, France; kliemannn@fellows.iarc.fr; 6 Cardiac Primary Prevention Research Center, Tehran Heart Center, Tehran University of Medical Sciences, Tehran 1416753955, Iran; farzad.masoudkabir@gmail.com; 7 Department of Cardiology, Tehran Heart Center, Tehran University of Medical Sciences, Tehran 1411713138, Iran; 8 Instituto Federal Goiano, Ceres 76300-000, Goiás, Brazil; 9 Hypertension Research Center, Cardiovascular Research Institute, Isfahan University of Medical Sciences, Isfahan 8158388994, Iran; nmohammadifard@gmail.com; 10 Isfahan Cardiovascular Research Center, Cardiovascular Research Institute, Isfahan University of Medical Sciences, Isfahan 8158388994, Iran; 11 School of Population and Public Health, Faculty of Medicine, University of British Columbia, Vancouver, BC V6T 1Z3, Canada

**Keywords:** visceral obesity, cancer, cardiovascular disease, pathophysiological mechanisms, pharmacological treatments

## Abstract

The association between obesity, cancer and cardiovascular disease (CVD) has been demonstrated in animal and epidemiological studies. However, the specific role of visceral obesity on cancer and CVD remains unclear. Visceral adipose tissue (VAT) is a complex and metabolically active tissue, that can produce different adipokines and hormones, responsible for endocrine-metabolic comorbidities. This review explores the potential mechanisms related to VAT that may also be involved in cancer and CVD. In addition, we discuss the shared pharmacological treatments which may reduce the risk of both diseases. This review highlights that chronic inflammation, molecular aspects, metabolic syndrome, secretion of hormones and adiponectin associated to VAT may have synergistic effects and should be further studied in relation to cancer and CVD. Reductions in abdominal and visceral adiposity improve insulin sensitivity, lipid profile and cytokines, which consequently reduce the risk of CVD and some cancers. Several medications have shown to reduce visceral and/or subcutaneous fat. Further research is needed to investigate the pathophysiological mechanisms by which visceral obesity may cause both cancer and CVD. The role of visceral fat in cancer and CVD is an important area to advance. Public health policies to increase public awareness about VAT’s role and ways to manage or prevent it are needed.

## 1. Introduction

Visceral obesity is a type of body fat deposition in the upper part of the body and within the abdominal cavity. This adipose tissue is located near several organs, such as the liver, stomach and intestines and it can build up in the arteries. Visceral fat is sometimes known as “active fat” because it can actively increase the risk of adverse health problems. Visceral adipose tissue (VAT) is a complex and metabolically active tissue, which can produce different adipokines and hormones responsible for endocrine-metabolic comorbidities. It is associated with increased adipocytokine production, proinflammatory activity and altered blood lipids levels as well as with decreased HDL cholesterol [1].

There is growing consensus that visceral obesity represents an important risk factor for diabetes, cardiovascular disease (CVD) and different types of cancers [2,3,4,5] such as esophagus, pancreas, colorectum, breast, endometrium, kidney and prostate [6,7]. A growing body of evidence suggests an overlap of the epidemiological risk factors of both CVD and cancer [5,8]. In fact, the association between obesity, cancer and CVD has been established in a multitude of animal and epidemiological studies [5,8]. Research on this topic is now focusing on the role of VAT on carcinogenesis and development of CVD, which may involve alterations in immunological, metabolic and endocrinal pathways.

Therefore, this review aims to highlight the potential shared disease pathways linking visceral obesity to cancer and CVD. In addition, we discuss the shared pharmacological treatments that may mitigate the risk of both conditions.

## 2. Pathophysiological Evidence of the Shared Role of Visceral Obesity in the Occurrence of Cancer and CVD

**Figure 1:**
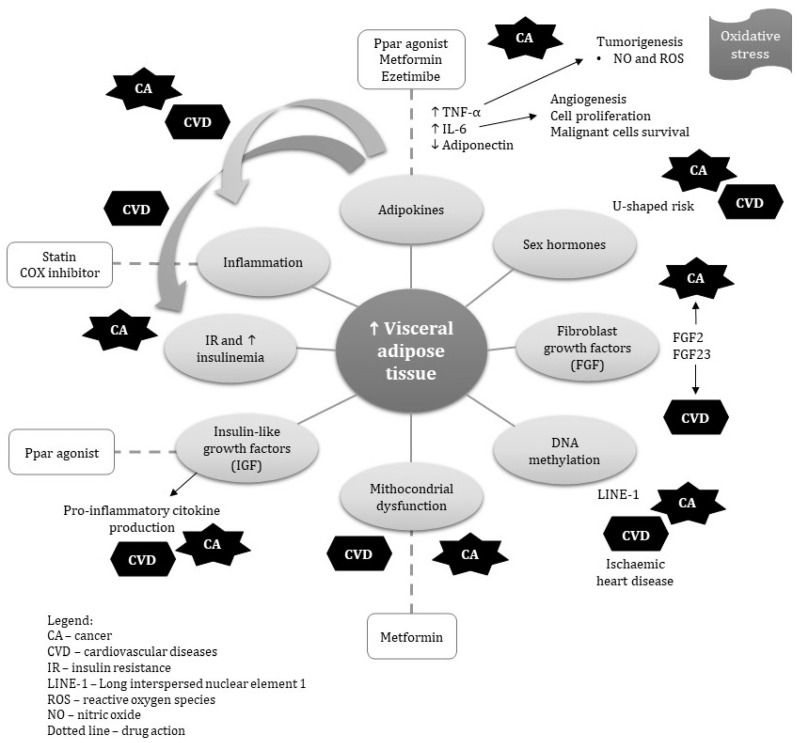
Summary of the main pathophysiological mechanisms shared in the development of cardiovascular disease (CVD) and cancer.

Figure 1 summarizes the main pathophysiological mechanisms shared in the development of CVD and cancer. In Table 1, we present a summary of the risk factors in visceral obesity, cardiovascular disease and cancer.

### 2.1. Inflammation

Increases in VAT cause adipose dysfunction and induce chronic local inflammation, which mediates most obesity-related complications [9]. The inflammatory process of the adipose tissue is characterized by the infiltration of classically activated M1 macrophages, leading to the production of reactive oxygen species (ROS) and release of pro-inflammatory cytokines such as interleukin 6 (IL-6) and tumor necrosis factor-α (TNF-α) [10].

Chronic inflammation has been linked to the development and progression of cardiovascular diseases [11] and several cancers [12,13], suggesting a shared pathophysiological role [14]. Regarding CVD, a chronic low-grade inflammatory environment is associated with the development of metabolic syndrome, type 2 diabetes, hypertension and dyslipidemia, which are linked to increased risk of cardiovascular events [11]. As for cancer, chronic inflammation is central for its development and progression, since inflammatory cells and mediators promote angiogenesis and sustain proliferative signaling [9,15,16,17]. Excessive caloric intake results in a dynamic response of adipose tissue via adipocyte hypertrophy and hyperplasia [18]. The rapid expansion of adipose tissue in obesity results in cellular hypoxia and activates hypoxia-inducible factor 1-α (HIF 1α) [19]. This, in turn, increases the expression of IL-6 and leptin, decreases adiponectin production and mediates the attraction of macrophages into the adipose tissue [20,21]. It has been reported that due to enhanced basal lipolysis [22], leakage of free fatty acids (FFAs) is increased in hypertrophic adipocytes. The released FFAs promote inflammation through binding to toll-like receptors 2 and 4 which results in activation of nuclear factor-kappa B (NF-κB) and c-Jun N-terminal kinase (JNK) signaling pathways [23]. The activation of aforementioned pathways increases the synthesis and secretion of pro-inflammatory cytokines such as IL-6.

Along with their role in chronic inflammation, IL-6 and TNF-α also contribute to other mechanistic effects on cancer development. Regarding IL-6, this adipocytokine stimulates angiogenesis, promotes cell proliferation, increases survival of malignant cells and inhibits cancer cell apoptosis, for example, in colon cancer [24,25]. As for the TNF-α, it is involved in all stages of tumorigenesis. TNF-α stimulates the growth and survival of malignant cells, promotes angiogenesis, invasion and migration of malignant cells, and suppress cytotoxic T lymphocytes and activated macrophages [26]. Moreover, TNF-α plays an important role in the initiation of tumors by stimulating the production of nitric oxide (NO) and ROS. ROS are well-known genotoxins [27], and it has been demonstrated that *Trp53^−/−^* in mice maintained in relatively hypoxic conditions (10% O_2_) have a significantly reduced level of tumorigenesis and improved survival compared to *Trp53^−/−^* mice maintained in standard atmospheric conditions (21% O_2_) [28]. Moreover, ROS trigger potentially oncogenic signal transduction cascades including mitogen-activated protein kinase (MAPK) and epidermal growth factor receptor (EGFR) signaling [29].

### 2.2. Adipokines

Adipokines are hormones secreted by the adipose tissue, such as adiponectin and leptin that regulate systemic metabolism and inflammation. They have been suggested as a link between obesity and other disorders such as cardiovascular disease and cancer [9]. Adiponectin has autocrine activity that results in adipocytes cell differentiation. In adipocytes, some factors such as sterol regulatory element-binding protein (SREBP)-1c promotes adipogenesis and enhances lipid content [30]. Excess weight gain may promote profound changes in the adipokines production increasing the risk of cancer and cardiovascular disease [9].

Adiponectin is a protein hormone with vasoprotective properties [31] and antineoplastic activity [32]. Clinical studies indicate that hypoadiponectinemia is associated with peripheral arterial dysfunction, hypertension, dyslipidemia and cancer initiation and poor prognosis [33,34]. Adiponectin inhibits ROS production as well as monocyte adhesion, which induces vasodilation. It also activates AMP kinase that leads to an increase in endothelial NO, synthase (eNOS) activity and NO production. The vascular system is protected by endothelial-derived NO, which enhances vasodilation and inhibits platelet aggregation, monocyte adhesion [35]. High glucose concentration induces production of ROS. However, adiponectin inhibits this process via cAMP/PKA-dependent pathway in endothelial cell [36].

Adiponectin attenuates the interaction between leukocytes and endothelial cells by suppressing the expression of E-selectin and vascular cell adhesion molecule-1. This adiponectin-related decrease in expression of adhesion molecules has been demonstrated in an animal model of atherosclerosis. Adiponectin inhibits the expression of adhesion molecules after induction by TNF-α and IL-8, which leads to reduction of monocyte attachment to endothelial cells [37]. Although adiponectin has numerous effects on the arterial wall, on the liver, as well as on insulin actions, its independent contribution to the etiology of CVD remains controversial as a systematic review and meta-analysis failed to identify it as an independent risk factor for cardiovascular outcomes [38]. Adiponectin plays a crucial mediator role in the pathogenesis of obesity-associated malignancies and its blood concentration reduces because of weight gain [34]. Clinical studies indicate that hypoadiponectinemia is associated with peripheral arterial dysfunction, hypertension, dyslipidemia and cancer initiation or progression [33,39]. Lower levels of adiponectin are also associated with poor colorectal and prostate cancer prognosis [34]. It is believed that adiponectin exerts its anticancer properties via direct and indirect mechanisms. It stimulates receptor-mediated signaling pathways and induces apoptosis. In an experimental study on HeLa cells by Xie et al. [40], it was observed that low adiponectin levels resulted in a significant increase in cell population in G0/G1 phase, concomitant with a reduction of cell number in S and G2/M phases which proves the inhibition of proliferation by adiponectin. In addition, they observed that adiponectin inhibited proliferation by downregulating cell cycle regulators such as cyclin D1 and c-myc and also activated apoptosis by inducing the expression of p21, p53 and Bax and the reduced level of Bcl-2 [41]. Recent studies by Mauro et al. also demonstrated that in breast cancer MDA-MB-231 xenograft models, the pre-treatment with adiponectin reduced tumor growth via amplifying AMP kinase signaling and reducing cyclin D1 expression [42,43]. Adiponectin may also act indirectly by modulating insulin sensitivity at the target tissue site, regulating inflammatory responses and influencing tumor angiogenesis. Adiponectin has a different isoform in different tissues and tumors, which may exert different effects on cancer initiation or suppression. Hence, the exact biological pathway linking adiponectin to cancer remains unclear and there are some controversial results [33].

Visfatin is another adipokine and cytosolic enzyme that was originally identified as pre-B cell colony-enhancing factor- 1 (PBEF) and has nicotinamide phosphoribosyl-transferase (Nampt) activity. Visfatin is predominantly produced in VAT. However, it is also produced by immune cells (e.g., neutrophils and macrophages) and induces expression of IL- 1b, TNF-α, and especially IL-6 in human leukocytes [44]. Over the last decade, visfatin has been suggested as a potential link between obesity and both cardiovascular disease and cancer [45]. It also induces matrix metalloproteinase (MMP)-9 and NF-κB expression in human endothelial cells, as they play a critical role in the pathophysiology of vascular inflammation in obesity and atherosclerotic plaque instability [46].

In addition to the potential indirect role of visfatin in cardiovascular disease through metabolic syndrome and inflammation, visfatin has been demonstrated to induce endothelial dysfunction in vivo, which has a pivotal role in initiation and progress of atherosclerosis [47]. Romacho et al. have recently reported that visfatin induces endothelial dysfunction in mice by a Nampt-dependent Toll-like receptor-4-mediated pathway, involving nod-like-receptor-protein-3 (NLRP3)-inflammasome and paracrine IL-1β [48]. Wang et al. demonstrated that visfatin also stimulates vascular smooth muscle cells proliferation via nicotinamide mononucleotide-mediated ERK1/2 and p38 signaling [49]. Other direct actions on atherogenesis via induction of angiogenesis, increased levels and activity of matrix metalloproteinases (MMP 2 and 9) as well as the promotion of cell adhesion molecules like the intercellular adhesion molecule 1 (ICAM-1), vascular cell adhesion molecule 1 (VCAM-1) and E-selectin have also been proposed [47,50]. All these actions, especially in combination with other risk factors, might result in increased risk of atherosclerotic cardiovascular disease initiation and progression.

The emerging evidence regarding the abnormal expression of visfatin in many types of cancers and also significant correlation between high circulating visfatin levels and increased risk of cancer and cancer-related mortality, suggests visfatin as a potential mediator that contributes to the interconnection of obesity and cancer [51]. Visfatin regulates the proliferation of cancer cells, as demonstrated in melanoma cells, where visfatin/Nampt induces proliferation and inhibits p53-dependent apoptosis via the E2F2/SIRT1 axis [52]. Similar action has been reported from visfatin in other cancer cell lines through PI3K/Akt and MAPK/ERK1/2 signaling pathways [51,53]. In addition to induction of tumor cell proliferation, visfatin induces angiogenesis through activation of the mTOR pathway, thereby increasing the expression of vascular endothelial growth factor (VEGF) and HIF-1α in endothelial cells [54]. Moreover, visfatin induces the expression of fibroblast growth factor (FGF)-2 gene in a Notch1-dependent manner, and triggers tube formation of endothelial cells [55].

### 2.3. Insulin, Insulin Resistance and Insulin-Like Growth Factors (IGF)

Visceral adiposity is also associated with metabolic dysfunction, increasing the risk of insulin resistance and hyperglycemia [9]. Several epidemiological studies indicate that insulin resistance and hyperinsulinemia are more important risk factors for some cancers and cardiovascular disease than obesity per se [56,57]. In terms of potential biological mechanisms, it has been suggested that the high concentrations of cytokines and low concentrations of adiponectin may interfere with glucose homoeostasis and lead to chronic hyperinsulinemia and insulin resistance [58]. Additionally, the rate of lipolysis is higher in VAT than subcutaneous adipose tissue, increasing the circulation of non-esterified fatty acids, which may affect hepatic insulin removal and lead to insulin resistance and hyperinsulinemia [59]. Insulin resistance decreases eNOS activation and NO production, which leads to the inflammation of plaque [58]. These steps sensitize plaque to rupture and consequently cause thrombotic vascular occlusion [60]. Several epidemiological studies have shown that insulin resistance is associated with an increased risk of breast, prostate and colon cancers [61].

Hyperinsulinemia may have carcinogenic effects via insulin receptors in the pre-neoplastic cells. It affects the insulin-like growth factors (IGF) axis, which leads to binding of IGF-1 or IGF-2 to the IGF-1 receptor promoting cell proliferation, differentiation and protection from apoptosis [62]. Epidemiological studies have shown that high endogenous or exogenous insulin release is associated with increased risk of colorectal, post-menopausal breast, pancreas and endometrium cancers [63]. 

IGF-1 level is significantly increased in individuals with obesity and this is a consequence of hyperinsulinemia inhibiting production of IGFBP-1 and -2 [64]. IGF-1 leads to macrophage migration and invasion and induces macrophage pro-inflammatory cytokines production. IGF-1 has pleotropic actions on heart and regulates contractility and heart apoptosis IGF-1 signaling. IGF-1 deficiency also increases the risk of cardiovascular disease, and IGF-1 receptor (IGF-1R) activation on heart protects it from effects of myocardial infarction [65]. Interestingly, high IGF-1 levels, common in patients with acromegaly, are associated with increased cardiovascular mortality too. Higher mortality from ischemic heart disease and stroke is seen in GH-deficient patients and/or low IGF-I levels, while high IGF-I levels induce cardiac hypertrophy and valve calcification [66]. IGF-1R activation promotes cell migration and affects E-cadherin and α- and β- catenin (cell adhesion molecule). In different meta-analyses [63], increased levels of IGF-1 were associated with risk of ovarian [67], colorectal, prostate [68] pre- and post-menopausal breast cancers [69]. However, similarly to the association between IGF-1 and CVD, recent findings suggest that both low and high levels of IGF-1 are associated with cancer mortality [70]. Therefore, IGF-I levels have a U-shaped relation with CVD and cancer. In conclusion, both low and high IGF-1 appear to be risk factors for increased cancer and cardiovascular disease incidence and mortality.

### 2.4. Sex Hormones and Lipid Profile

Visceral fat mass is lower in female than males, but this difference is diminished in elderly people. Therefore, it seems that estrogen signaling plays a major role in sex differences in adiposity [71]. Clinical studies suggest that CVD risk is higher in males than in premenopausal females, regardless of body size. Plasma HDL cholesterol levels are significantly higher and fasting plasma glucose concentrations significantly lower in women compared to men who differ in VAT. This suggests that VAT is an important correlate of the gender differences observed in CVD risk [72]. In men, low circulating levels of total testosterone and sex hormone-binding globulin (SHBG), a determinant of testosterone bioavailability, are generally associated with abdominal and/or visceral obesity and affect metabolic syndrome [73].

Clinical studies have provided strong support regarding the association between sex hormones, obesity and cancer, specifically for hormone-dependent cancers such as endometrial, breast, uterine, ovarian, and prostate cancers [74] and for multiple myeloma and non-Hodgkin lymphoma [75]. Adipocytes influence sex hormone biology by increasing aromatase activity and consequently the production of estrogen in post-menopausal women, and of testosterone in men [76]. Estrogen is a well-known promoter of tumorigenesis through oncogenic transcriptional regulation of genes involved in cell survival and proliferation [77], and non-genomic crosstalk with growth factor pathways, including epidermal growth factor (EGF), IGF, and FGF [78]. Estrogen signaling disturbance plays a crucial role in the development of mammary tumors. Exogenous or parity-associated excessive estrogen supply is suppressive against triple negative but not ER-positive breast cancer, which reduces the ER expression of tumors and reduces the anticancer capacity. It seems that ER-positive or negative breast cancers have a different mechanism [79].

### 2.5. Fibroblast Growth Factor

FGFs comprise a broad family of polypeptides with significant biological roles. They are signaling proteins related to development and metabolism, covering 22 members in the mammalian FGF family. Secreted FGFs control important cellular processes that comprehend positive and negative regulation of proliferation, survival, migration, differentiation, and metabolism. FGFs may indirectly contribute to an inflammatory environment through the synergistic potentiation of inflammatory mediator-induced leukocyte recruitment, intensifying cell adhesion and molecule upregulation [80]. Some FGFs play a pathophysiological role in cardiovascular diseases [81] and in the development of various types of cancers [82].

In cardiovascular disease, some FGFs are implicated in cardiac remodeling through different mechanisms, which can contribute to heart failure [81]. For example, FGF2 induces cardiac hypertrophy through the activation of FGF receptor 1c and MAPK signaling, and FGF23 by activating calcineurin/NFAT signaling without αKlotho [81]. In tumor growth, the FGF receptor system plays important roles. The FGFR-1-mediated MAPK kinase pathway has been implicated in tumor cell proliferation. In skin carcinoma and oral carcinomas, FGFR-2 expression has been found to be associated with early tumor development.

Although FGFs are implicated in both cardiovascular disease and cancer development, different FGF family members are involved in the pathophysiology of these illnesses. In an experimental model with rats, fibroblast growth factor 2 (FGF2), secreted and released by the VAT, stimulated the transformation of skin epithelial cells, primarily in mice fed with a high-fat diet (60% kcal from fat) [83]. This study also found that signaling through the tyrosine kinase FGF2 receptor-1 (FGFR1) plays an important role in FGF2-stimulated transformation, i.e., post-initiation phases a cell undergoes to become malignant [83]. Besides skin cancer, the FGF2–FGFR1 axis was also implicated in a VAT-stimulated transformation of breast epithelial cells [84]. Regarding cardiovascular diseases, we highlighted the role of FGF23. Although not secreted by the VAT, a recent study found FGF23 levels to be elevated among men and post-menopausal women with obesity, mainly those with abdominal obesity, suggesting an association with visceral fat accumulation [85]. Several studies have found an independent association of serum levels of FGF23 and heart disease [86,87,88,89,90].

### 2.6. Alterations in DNA Methylation

Changes in DNA methylation is one of the most studied epigenetic modifications at the molecular level [91]. DNA methylation consists of the addition of a methyl group in the position 5 of the DNA cytosine ring through DNA methyltransferases enzymes [92]. Long interspersed nuclear element 1 (LINE-1) has been implicated as a marker of the global DNA methylation of the genome [93]. The LINE-1 is usually heavily methylated and its hypomethylation has been associated with cancer development and progression [31]. Additionally, LINE-1 methylation variability has been linked with ischemic heart disease and stroke, suggesting they share a pathophysiological role between cancer and cardiovascular diseases.

DNA methylation in visceral adipose tissue has also been the subject of studies in recent years, given that it is an important mechanism that predisposes the development of obesity and metabolic impairment [94,95]. In a study of severely obese individuals, lower overall DNA methylation, assessed by analysis of LINE-1 in visceral adipose tissue, was associated with an increased risk of metabolic syndrome [96]. Hypomethylation of DNA may be a mechanism shared between metabolic changes in visceral adipose tissue and cancer.

### 2.7. Other Biochemical and Metabolic Factors

Mitochondrial dysfunction can promote various mechanisms during cancer progression. Mitochondrial alterations affect nuclear gene expression for neoplastic transformation. Alteration in the intracellular level of oncometabolites play a critical role in neoplastic transformation and cancer progression. In addition, other suppressors such as hypoxia-inducible factor 1 and p53, alter cellular metabolism [97]. Impairment of mitochondrial function in VAT was found to be linked to esophageal adenocarcinoma [98]. It has been shown in an in vitro study, that the adipose media of patients with obesity and esophageal adenocarcinoma induces mitochondrial dysfunction which in turn can promote carcinogenesis [98]. On the other hand, in an animal model, mice deficient in mitochondrial transcription factor A (TFAM) in adipocytes, one of the main regulators of mitochondrial mass and function, presented mitochondrial dysfunction that led to lipoatrophy, insulin resistance, hepatosteatosis, hypertension and cardiac dysfunction [99]. Additionally, lipodystrophy syndrome has been associated with insulin resistance and cardiovascular complications [99].

## 3. Pharmacological Treatment of Visceral Obesity

The development of pharmacological agents to reduce visceral obesity is difficult due to various potential side effects [100]. For example, regulatory authorities in visceral obesity and weight loss had approved dexfenfluramine, sibutramine, and rimonabant. However, they were removed from clinical use because of their various side effects. In this section, we introduce the clinical and experimental drugs that can probably reduce visceral fat that affects the risk of cancer and CVD. The summary of the effects from pharm therapy on cancer and CVD is presented in Table 2.

### 3.1. Peroxisome Proliferator-Activated Receptor Gamma (PPARγ)

This ligand-activated transcription factor has critical roles in various cellular functions and glucose and lipid metabolism [101]. PPARγ is found in abundance in the adipose tissue where this receptor acts as a lipid sensor. The activation of PPARγ (by its ligands) leads to adipokine secretion [102] which reduces visceral fat, but not necessarily body weight. It has been suggested that pioglitazone (PPARγ agonists) decreases visceral fat, however, it may increase total body weight [103]. PPARγ agonists have cardiovascular protective effects, but some adverse cardiovascular events such as congestive heart failure and myocardial infarction have been detected with these drugs. For example, pioglitazone showed a reduction in the risk of MI (miocard infarction), stroke or death in patients with type 2 diabetes mellitus [104] in a recent meta-analysis. However, another meta-analysis showed that rosiglitazone increased the risk of MI but with low mortality risk [105].

PPAR-γ agonists decrease tumor proliferation by lowering circulating insulin and affecting key pathways of the Insulin/IGF axis, such as PI3K/mTOR, MAPK, and GSK3-β/Wnt/β-catenin cascades, which regulate cancer cell survival, cell reprogramming, and differentiation [106]. One meta-analysis supported a protective association between PPAR-γ agonists’ use and reduction of colon cancer risk in patients with DM [107]. On the other hand, in human studies, it has been shown that Pioglitazone can increase the risk of bladder cancer in humans [108] depending on its dosage. While Pioglitazone decreases the risk of breast cancer. The other PPAR agonist, rosiglitazone, decreased [109] the risk of bowel cancer significantly. Therefore, the effect on cancer may be site and drug specific.

### 3.2. Growth Hormone Treatment

Growth hormone (GH) treatment is not effective in treating visceral obesity in patients with normal levels but its deficiency leads to obesity or visceral obesity [110]. It has been suggested that GH therapy decreases visceral adiposity and improves lipid profile in adults with obesity [111]. This effect results from GH lipolysis properties [112]. However, IGF-I and IGFBP-3 levels are more related to visceral adipose tissue accumulation than overall adiposity [113].

Recombinant human growth hormone (rhGH) has been widely used to treat children with short stature secondary to any medical problems. The experience from many thousands of patients and years of treatment demonstrates a good safety record for rhGH. Nevertheless, the findings from a meta-analysis showed a significant increase in all-cause mortality but no significant increase in the malignancy and CVD mortality. The risk for second neoplasms increases in these patients [114].

A recent meta-analysis evaluated the risk of cancer in adults with and without growth hormone replacement therapy, which suggested that growth hormone replacement therapy could reduce the risk of cancer in adults with growth hormone deficiency [115].

### 3.3. Metformin

Metformin, a biguanide anti-hyperglycemic agent, is the first line treatment for overweight diabetes patients [116]. It reduces liver glucose production, increases cells insulin sensitivity and induces anorexia effect [117]. Metformin treatment up-regulates adipose oxidation-related enzymes in the liver and also UCP-1 in the brown adipose tissue which leads to reductions in abdominal obesity in mice [118]. In addition, metformin can reduce visceral adiposity by upregulating adaptive thermogenesis [119].

Metformin has protective effects on cardiovascular problems in patients with type 2 diabetes which are independent from the glucose-lowering effect [120]. Metformin activates AMPK promoting glycolysis. Metformin also increases eNOS production, which results in beneficial effects in patients with heart failure [121]. Metformin attenuated ER stress-induced mitochondrial dysfunction in myocardial cells, which results in reduction of cardiac injury through ER-stress [122].

Metformin increases glucose uptake and insulin sensitivity and reduces serum insulin level which results in reduction of pre-neoplastic and neoplastic cell proliferation [123]. Metformin also reduces circulating levels of androgen and estrogen, which is another potential mechanism of metformin in the prevention of cancer incidence [124]. Metformin inhibits mTOR activity and activates p53 which reduces the cell cycle. Metformin reduces mortality risk and recurrence of cancers in clinical studies and also sensitizes cancer cells to chemo and radiotherapy [123]. The most recent meta-analysis confirms the association between metformin use and reduction of pancreatic and colorectal cancer incidence [63,125].

### 3.4. Cycloxyganase Inhibitors

Elevated levels of cyclooxygenase (COX), a sign of chronic inflammation, is the key connection between cancer and obesity. Non-steroidal anti-inflammatory drugs (NSAIDs) reduce inflammation via COX inhibition which ultimately reduces prostaglandin levels [126]. They also have a different effect on visceral obesity and adipose reduction [127].

The results from randomized controlled trials (RCTs) and meta-analyses show that there is a significant increase in risk of heart attack in patients who are taking COX inhibitors compared with placebo [128]. The possible mechanisms are due to the fact that prostacyclin reductions lead to platelet aggregation and vasoconstriction [129]. Cancer cells and tissues upregulate the expression of COX which is inversely associated with cancer incidence and recurrence [130]. It has been confirmed that COX inhibitors can reduce the risk of breast cancer in women [131]. However, because of adverse cardiovascular effects, they are not currently prescribed for prevention of cancer risk and recurrence.

Aspirin, an irreversible inhibitor of the COX enzyme [132], is an exception, which has protective effects on the cardiovascular system. It has been confirmed that low-dose aspirin use may reduce the risk of cancer. These observations coincide with recent in vivo and clinical studies showing a functional relationship between platelets and tumors, suggesting that aspirin’s chemo preventive properties may result, in part, from direct modulation of platelet biology and biochemistry [133].

### 3.5. Statins

Statins, HMG-CoA reductase inhibitors, are the most common lipid-lowering medications and are widely prescribed. They are effective in lowering cholesterol and play a critical role in the prevention of primary and secondary cardiovascular disease [134]. Statins also decrease chronic inflammation and oxidative stress, which is another possible mechanism in preventing cardiovascular disease [135]. It seems that some types of statins, such as pitavastatin may increase circulating adiponectin. However, data are conflicting [136]. A recent study confirmed that atorvastatin and rosuvastatin (two other statins) reduce epicardial adipose tissue in post-menopausal women independent of their lipid-lowering property [137]. However, it seems that different types of statins may have different effects.

There are several meta-analyses on the effect of statins and cancer incidence and also relapse with some showing beneficial effects while others found no effects. A risk reduction has been observed in esophageal [138], colorectal [139] and gastric cancer [140]. It seems that they may reduce the risk of mortality from cancer too [141,142].

### 3.6. Ezetimibe

Ezetimibe is another lipid-lowering drug which limits the absorption of cholesterol from the gastrointestinal tract epithelial. It blocks the Niemann-Pick C1-like 1 (NPC1L1) protein on small intestine epithelial cells, which leads to reduction of plasma level of cholesterol [143].

There is some evidence suggesting that ezetimibe has an effect on adiponectin and decreases insulin resistance which results in adipose tissue reduction in patients with metabolic syndrome [144]. Adding ezetimibe to statin therapy is associated with a greater reduction in blood level of TNF-α in patients with hyperlipidemia [145].

Ezetimibe in combination with statin, has beneficial effects on the risk reduction of non-fatal MI. However, there is limited evidence about its monotherapy and CVD risk reduction [146]. It has been shown that ezetimibe could suppress inflammation and liver tumor growth in animal models of a high fat diet. It seems that inhibiting angiogenesis in mice leads to tumor suppression [147]. However, cholesterol lowering by ezetimibe did not slow prostate tumor growth and may induce expression of LDL receptor in cancer cells [148].

## 4. Conclusions

In this review, we discussed some pathophysiological aspects shared between VAT, cardiovascular disease and cancer as well as their shared pharmacological prevention. Chronic inflammation and dysregulated metabolism associated to visceral obesity, such as insulin resistance, hyperglycemia, and dyslipidemia can affect both CVD and cancer development and progression. The shared disease pathways linking visceral obesity to cancer and CVD may offer valuable opportunities for public health interventions to tackle both diseases. Reductions in abdominal and visceral adiposity improve insulin sensitivity, lipid profile and cytokines, which consequently reduce the risk of CVD and some cancer types. In fact, in addition to lifestyle interventions, pharmacotherapy with antidiabetic drugs, PPARγ and recombinant growth hormone may decrease the risk of both visceral obesity and cancer. Several medications have been shown to reduce visceral and/or subcutaneous fat. However, none of them have been approved for use in this context. Further research on the shared pathophysiological mechanisms underlying the association between visceral obesity, CVD and cancer could potentially lead to the discovery of important biomarkers and pathways and development and assessment of effective therapies. New pharmacological treatments that selectively affect the shared pathways, are needed. Long term, well designed RCTs and cohort studies should be conducted to test the hypothesis of targeting shared mechanisms aiming to prevent VAT, cancer and CVD. The role of visceral fat in cancer and CVD is an important area to advance in public health policies. It is also important to increase public awareness about its role and ways to manage or prevent it.

## Figures and Tables

**Table 1:** Risk factors in visceral obesity, cardiovascular disease and cancer.

Risk factor	Visceral Obesity	Cardiovascular Disease	Cancer
Inflammation	Reactive oxygen species (ROS) and release of pro-inflammatory cytokines such as interleukin 6 (IL-6) and tumor necrosis factor-α (TNF-α)	Metabolic syndrome, type 2 diabetes, hypertension and dyslipidemia,	Promote angiogenesis and sustain proliferative signaling
Adipokines	Adipocytes cell differentiation	Effect on nitric oxide synthase (NOS) and ROS, peripheral arterial dysfunction, hypertension, dyslipidemia	Proliferation and inhibits p53-dependent apoptosis
Insulin and insulin like growth hormone	Metabolic dysfunction,	Decrease eNOS activation and NO production, which leads to entrance of inflammation to plaque	Promote cell proliferation, differentiation and protection from apoptosis
Sex hormones	Estrogen signaling	Plasma HDL cholesterol levels are significantly higher and fasting plasma glucose concentrations	Increase aromatase activity
Fibroblast growth factor	Inflammation	Cardiac hypertrophy through the activation of FGF receptor	Transformation of epithelial cell
Alterations in DNA methylation	Long interspersed nuclear element activation	Metabolic syndrome, Ischemic heart disease	Cell proliferation

**Table 2:** Drugs for joint pharmacologic prevention of visceral obesity, cardiovascular disease and cancer.

Drugs	Direct Target	Action on Visceral Obesity	Action on Cancer	Action on CVD
PPARγ agonists	Peroxisome proliferator-activated receptor Gamma	Adipokine /IGF	Reduce circulating insulin, Reduce Angiogenesis Increase Apoptosis	Reduce blood pressure Reduce myocardial infarction and stroke Improve endothelial function
Recombinant human growth hormones	Growth hormone	Increase lipolysis/IGF-I	Reduce risk of cancer in adults with growth hormone deficiency	Reduce risk of MI in adults with growth hormone deficiency
Metformin	Unknown	Induces anorexia, upregulates adaptive thermogenes, and modulates adipokines Improves mithocondrial dysfunction	Reduces pre-neoplastic and neoplastic cell proliferation Reduces circulating levels of androgen and estrogen	Increases eNOS production Attenuates ER stress-induced mitochondrial dysfunction Reduces cardiac injury through ER-stress
NSAIDs	Cycloxyganase	Reduce prostaglandin levels Modulate adiponectin	Reduce inflammation	Reduce platelet aggregation and vasoconstriction
Statins	HMG-CoA reductase	Increase circulating adiponectin	Tumor-suppressor Increase Apoptosis	Improve endothelial function Plaque stabilization
Ezetimibe	Niemann-Pick C1-like 1 blocker	Modulate adiponectin Decrease insulin resistant which results	Suppress inflammation Inhibiting angiogenesis	Reduces in blood level of TNF-α in patients with hyperlipidemia
